# Adipose-derived stem cells promote tumor initiation and accelerate tumor growth by interleukin-6 production

**DOI:** 10.18632/oncotarget.3481

**Published:** 2015-03-08

**Authors:** Hong-Jian Wei, Rong Zeng, Jui-Hua Lu, Wen-Fu T. Lai, Wei-Hong Chen, Hen-Yu Liu, Ya-Ting Chang, Win-Ping Deng

**Affiliations:** ^1^ Graduate Institute of Biomedical Materials and Engineering, College of Oral Medicine, Taipei Medical University, Taipei, Taiwan; ^2^ Stem Cell Research Center, Taipei Medical University, Taipei, Taiwan; ^3^ Department of Orthopedic Surgery, The Affiliated Hospital of Guangdong Medical College, Zhanjiang, China; ^4^ Graduate Institute of Clinical Medicine, Taipei Medical University, Taipei, Taiwan

**Keywords:** Adipose-derived stem cells, Tumor initiation, Cancer stem cell, Breast cancer, Colon cancer

## Abstract

Adipose-derived stem cells (ADSCs) are multipotent cells that have attracted much recent attention. Here, we show that ADSCs enhance sphere formation and in vivo tumor initiation of breast and colon cancer cells. In co-culture, ADSCs induced several stem cell markers in cancer cells. ADSCs also accelerated tumor growth. Interaction of ADSCs and cancer cells stimulated secretion of interlukin-6 in ADSCs, which in turn acted in a paracrine manner on cancer cells to enhance their malignant properties. Interleukin-6 regulated stem cell-related genes and activated JAK2/STAT3 in cancer cells. We suggest that ADSCs may enhance tumor initiation and promotion.

## INTRODUCTION

Mesenchymal stem cells (MSCs) are multipotent cells that can self-renew and differentiate into various somatic lineages such as bone, adipose, cartilage, and muscle [1]. The natural ability of MSC is to repair dead or damaged tissues. Thus, these cells have shown great promise as a therapeutic approach in tissue engineering and regenerative medicine. MSCs were first identified and isolated from bone marrow [2] and subsequently proved to exist in a variety of tissue niches in the body such as adipose, muscle, tendon, umbilical cord blood, and amniotic fluid [3]. MSCs isolated from different origins with variable growth potential, but share similar surface markers and multilineage differentiation potential. Adipose tissue is one of the richest sources of adult stem cells. Compared to other MSCs, adipose-derived stem cells (ADSCs) are easier and less expensive to obtain [4]. Thus, ADSCs have attracted much recent interest due to their convenient acquisition and regenerative capability. ADSCs have emerged as therapeutic approaches in several medical fields such as plastic, orthopedic, and cardiac surgery as well as breast reconstruction.

Tumor-specific tropism is an important characteristic of MSCs. Previously, we have determined that bone marrow-derived MSCs could home to and engraft into tumor lesions via intravenous administration. After homing, the administered MSCs could differentiate into vascular endothelial and stromal cells to form the vessels and stroma of tumors [5]. Like other MSCs, ADSCs reveal tropism to inflammatory sites or tumor lesions and contribute to the tumor microenvironment [6-7]. Although current knowledge of the biological impacts of MSCs on tumor development is greatly improved, the underlying effects of MSCs from different origins remain controversial. Accumulating evidence indicates that ADSCs promote the tumor growth and metastasis of various cancers [8-11], whereas conflicting reports reveal the anti-tumorigenic potentials of ADSCs such as anti-proliferation and pro-apoptosis [12-14]. Hence, the elusive role of ADSCs in tumor development causes the safety concerns in clinical utilization. On the other hand, obesity is the excessive accumulation of fat tissue and associated with various diseases, including cancer. Obesity or overweight are associated with the incidence of several cancers such as breast and colon cancers [15]. Recent reports indicate that obesity accompanies ADSC expansion, including enhanced proliferation and increased number of ADSCs in fat tissue [16-17]. Moreover, Strong *et al*. demonstrated that obesity promotes significant changes in the biological properties of ADSCs and that these alterations enhance breast cancer tumor growth [18]. Hence, adipose tissue adjacent to tumor is thought to provide ADSCs to affect tumor progression, which may be the alternative mechanism of cancer incidence associated with obesity.

It is widely recognized that tumor development is a multistep process and can be divided conceptually into three steps consisting of tumor initiation, promotion, and progression. Among these three steps, ADSCs have been reported to affect tumor promotion (e.g., increased tumor growth) [19-21] and progression (e.g., enhanced metastasis) [9, 21-22], but their effects on tumor initiation have not been studied extensively. Hence, we purpose to investigate the functional roles of ADSCs in tumor development, especially in the early phase of tumorigenesis. We focus on breast and colon cancers herein. Breast cancer and colon cancer are serious public health problems worldwide with nearly 1.7 million and 1.4 million new cases in 2012 respectively [23]. Furthermore, breast and colon cancer incidence is shown to related to obesity [24-25]. In this study, primary ADSCs were isolated from the abdominal fat of mice and characterized with MSC-specific surface markers. Breast and colon cancer cells were then utilized to interact with ADSCs. We demonstrated that ADSCs play pro-malignant roles in both breast and colon cancer cells, including enhanced tumor initiation and accelerated tumor growth. Our results suggested the pro-malignant effects of ADSCs may be mediated by activation of interleukin-6 (IL-6)-related pathway.

## RESULTS

### ADSCs display MSC surface markers and multipotent differentiation capacity

**Figure 1:**
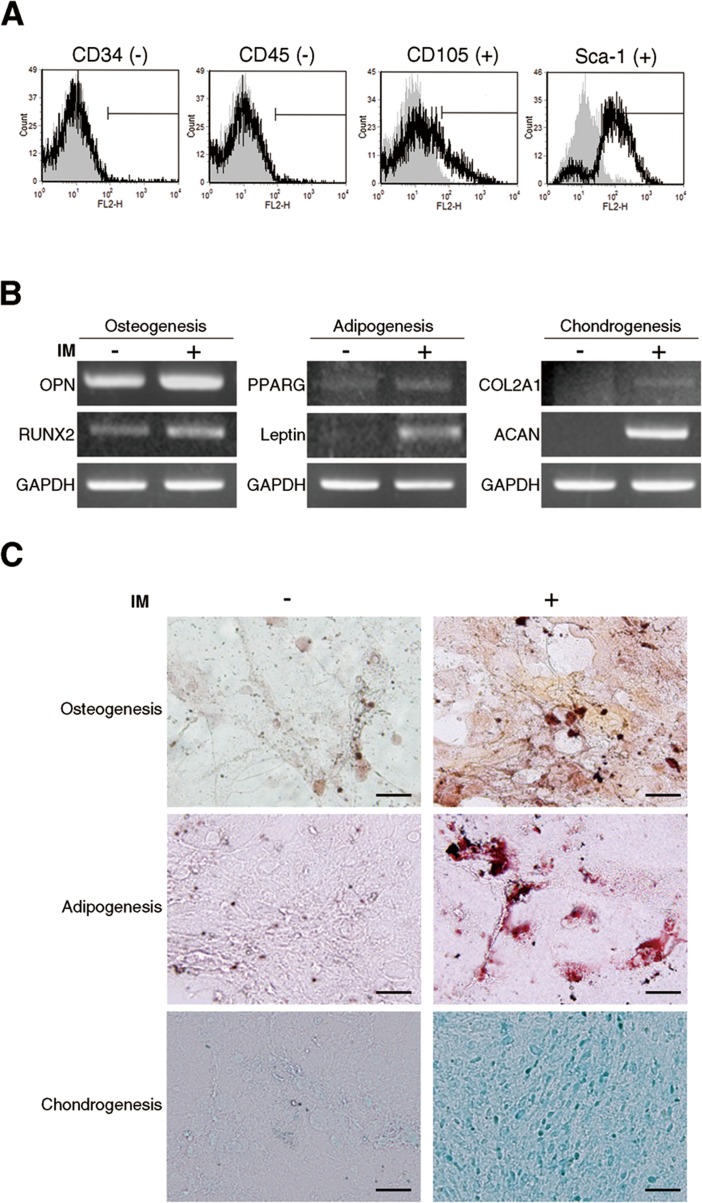
Characterization of ADSCs from mouse abdominal adipose tissues (A) Cell-surface marker profiles of ADSCs determined by flow cytometry using antibodies against indicated antigens; grey regions represent isotype controls. Multilineage differentiation capacity of ADSCs was identified by (B) specific marker gene expression and (C) lineage-specific staining. Osteogenic differentiation was assessed by Alizarin Red S staining for mineral nodule deposition. Adipogenic differentiation was assessed by Oil Red O staining for lipid vesicle formation. Chondrogenic differentiation was assessed by Alcian blue staining for proteoglycan deposition. IM: induction medium.

Adipose tissue is considered a source of adult stem cells of mesenchymal lineage. To determine whether stromal cells isolated from mice abdominal fat exhibited stem cell properties and were ADSCs, we conducted assays for cell surface markers and multilineage differentiation. As shown in Figure 1A, the cell surface marker profiles of isolated cells revealed similar immunophenotypes to MSCs, which were negative for CD34 and CD45 (hematopoietic stem cell markers) and positive for CD105 and Sca-1 (MSC markers). A key characteristic of MSCs is multipotency. We therefore tested whether the isolated cells displayed multipotent differentiation capacity including osteogenic, adipogenic, and chondrogenic differentiation. These cells were cultured under standard induction conditions, and *in vitro* differentiation was monitored by specific gene expression and lineage-specific staining. RT-PCR analysis demonstrated that upon induction the isolated cells upregulatd the differentiation marker genes of three different lineages. These differentiation marker genes are *OPN* and *RUNX2* for osteogenesis, *PPARG* and *Lepti*n for adipogenesis, and *COL2A1* and *ACAN* for chondrogenesis (Figure 1B). Parallel to gene expression results, lineage-specific staining showed that Alizarin Red S staining for osteogenic matrix, Oil Red-O staining for lipid droplet, and Alcian Blue staining for proteoglycan accumulation were strongly enhanced in isolated cells after induction (Figure 1C). These results indicate that cells derived from adipose tissue conserve key MSC characteristics, including specific surface markers and multipotent differentiation capacity, and are known as ADSCs.

### ADSCs enhance sphere generation, cancer stem cell marker expression, and *in vivo* tumor formation of breast and colon cancer cells

**Figure 2:**
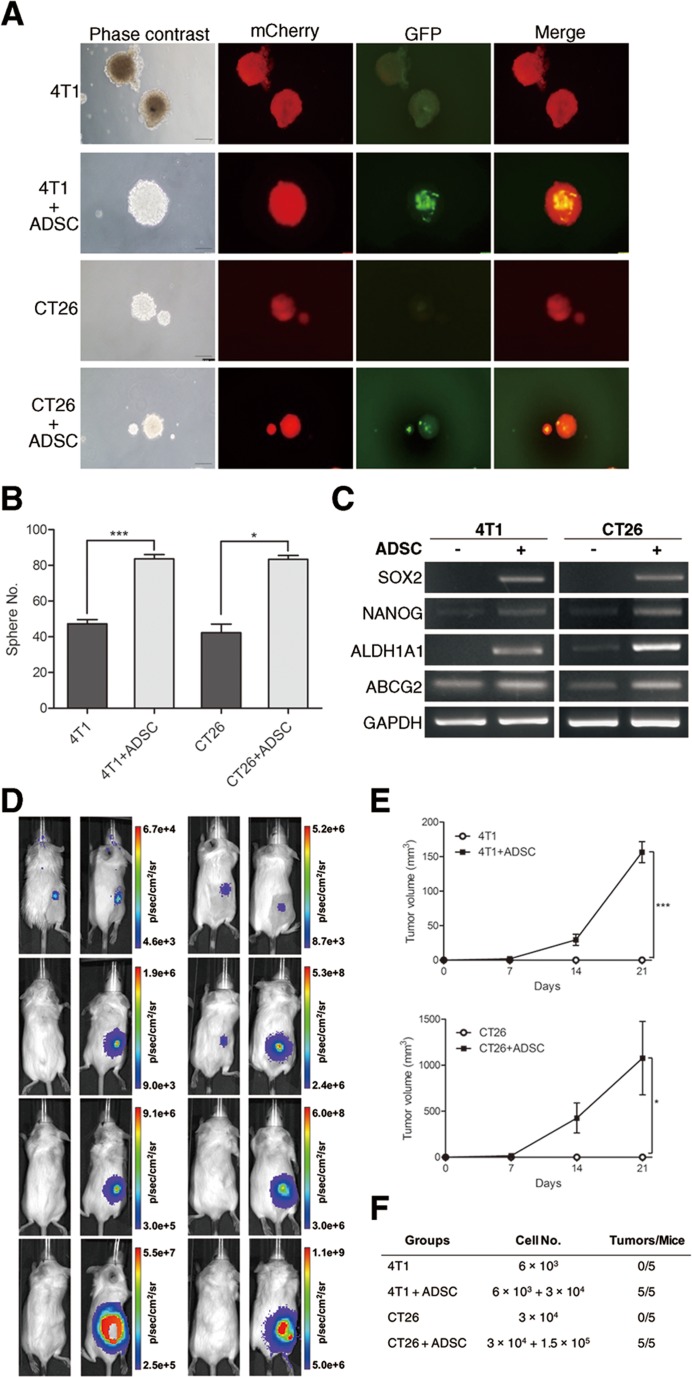
Enhanced tumor-initiating properties of breast and colon cancer cells by ADSC stimulation (A) Representative phase-contrast and fluorescence images and (B) quantitation of spheres generated by 4T1, 4T1 plus ADSCs, CT26, and CT26 plus ADSCs; scale bars indicate 100 μm. Values are means + SEM; *, P<0.05; ***P<0.001 in unpaired t test with Welch's correction. (C) mRNA expression of CSC markers *SOX2*, *NANOG*, *ALDH1A1*, and *ABCG2* were evaluated by RT-PCR; *GAPDH* served as loading control. (D) Representative bioluminescence images and (E) tumour volume measurements (means ± SEM) from syngeneic tumor models. Results were taken 0, 7, 14, and 21 days after subcutaneous injection of 4T1 or CT26 cells with or without ADSCs; *, P<0.05; ***, P<0.001 using two-way ANOVA. (F) Quantitation of tumor formation by 4T1 and CT26 cells with or without ADSCs in mice. Animals were implanted with indicated cell amounts subcutaneously, and the number of mice with tumors after 60 days is indicated.

Tumor development is thought to be a multistage progress, including tumor initiation, promotion, and progression. Cancer stem cells (CSCs) are a small population of cancer cells with stem-like properties. CSCs perform a critical role during tumor development, especially in tumor initiation. Thus, the properties of CSCs are highly associated with cancer incidence and poor prognosis of patients. Sphere formation assay has been extensively utilized to retrospectively recognize CSCs based on their reported ability to evaluate self-renewal at the single-cell level *in vitro* [26]. To investigate whether the tumor-initiating ability of breast and colon cancer cells was affected by ADSCs, we first performed tumor sphere assay. We utilized cancer cells transduced with mCherry fluorescent protein and ADSCs isolated from green fluorescent protein (GFP)-transgenic mice. We found that breast or colon cancer cells cultured alone were able to form 3-dimensional tumor spheres and, as expected, ADSCs alone showed no sphere generation. In co-culture, representative images showed that ADSCs could survive and integrate into breast or colon cancer spheres (Figure 2A). We found that the sphere-forming efficiency of breast or colon cancer cells was significantly increased while directly co-cultured with ADSCs (Figure 2B). RT-PCR analysis further revealed that cancer cells upregulate several CSC markers upon co-culture with ADSCs, including *SOX2*, *NANOG*, *ALDH1A1*, and *ABCG2* (Figure 2C). To evaluate whether *in vivo* tumor initiation of cancer cells was influenced by ADSCs, we subcutaneously inoculated 4T1 or CT26 cells with or without ADSCs into BALB/c mice. We then monitored tumor formation in mice by using non-invasive bioluminescent imaging. Representative images are shown in Figure 2D, and quantitative results are shown in Figure 2E and 2F. We found that ADSCs can markedly induce the formation of 4T1 and CT26 tumors, while cancer cells or ADSCs alone formed no tumors in mice. Above results indicate that ADSCs enhance the tumor-initiating properties of breast and colon cancer cells.

### ADSCs accelerate growth of breast and colon cancer cells

**Figure 3:**
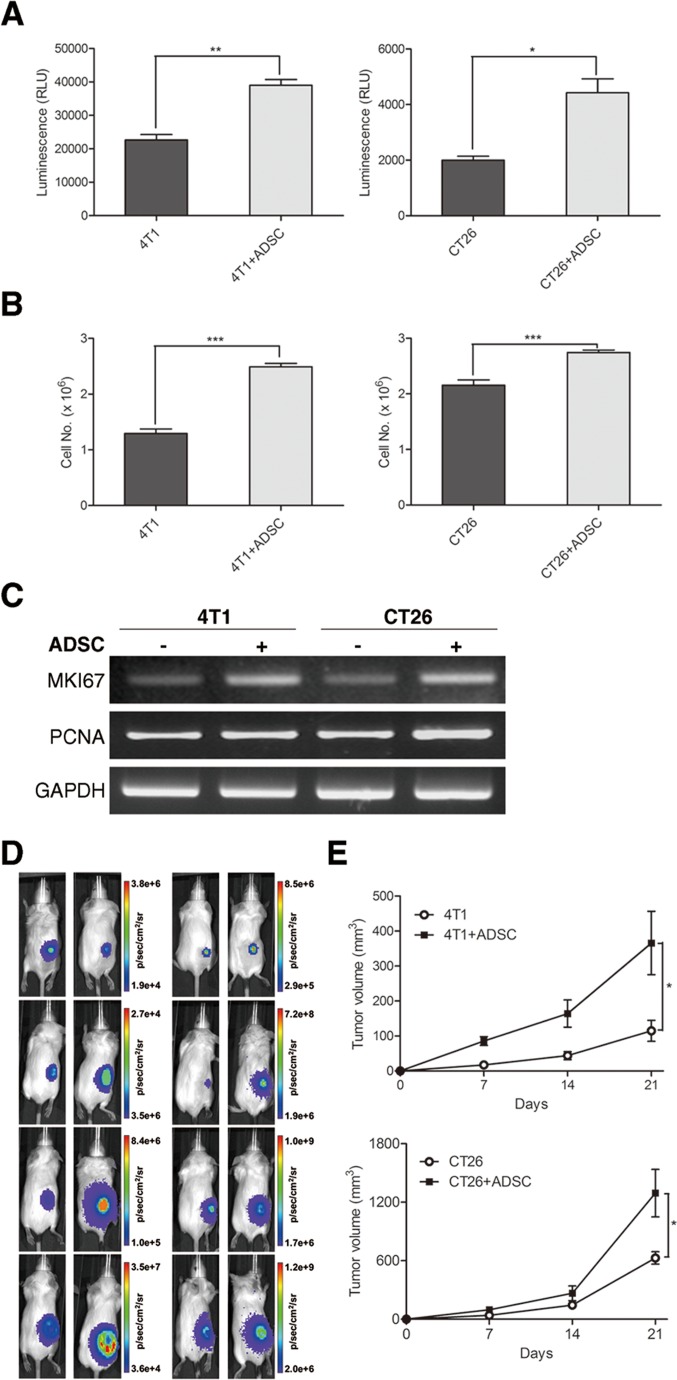
ADSCs accelerate breast and colon cancer cell growth both in vitro and *in vivo* (A) In vitro cell growth of 4T1 and CT26 cells co-cultured directly with or without ADSCs assessed by bioluminescence assay. Values are means + SEM; *, P<0.05; **, P<0.01 in unpaired t test with Welch's correction. (B) *In vitro* cell growth measured by cell counting of 4T1 and CT26 cells co-cultured indirectly with or without ADSCs using trans-well. Values are means + SEM; *** indicates P<0.001 using unpaired t test with Welch's correction. (C) RT-PCR of *MKI67* and *PCNA* expression in 4T1 and CT26 cells, with *GAPDH* as loading control. (D) Representative bioluminescence images and (E) tumour volume measurements (mean ± SEM) of 3 × 10^5^ 4T1 cells or 1.5 × 10^5^ CT26 cells injected subcutaneously into syngeneic mice alone with or without five folds of ADSCs. Results were taken 0, 7, 14, and 21 days after implantation; *, P<0.05 in two-way ANOVA.

To investigate whether the cell growth of breast and colon cancer cells was influenced by ADSCs, we directly co-cultured ADSCs with 4T1 or CT26 cells. The amount of cancer cells was evaluated by *in vitro* bioluminescent quantification. The bioluminescence activity was strongly enhanced in cancer cells co-cultured with ADSCs compared to cancer cells alone (Figure 3A), suggesting that ADSCs could increase the number of both cancer cells. ADSCs are known as a rich source of cytokines and chemokines, which can communicate with other surrounding cells in a paracrine manner. To further determine whether ADSCs enhanced cancer cell growth via paracrine effect, we co-cultured cancer cells with ADSCs indirectly using trans-well co-culture system. Consistent with above result, ADSCs could increase the cell number of breast and colon cancer cells upon co-culture with them (Figure 3B). We further found that ADSCs could upregulate the mRNA expression of proliferation-related genes in cancer cells, including *MKI67* and *PCNA* (Figure 3C). We then utilized syngeneic tumor models to evaluate whether the *in vivo* tumor growth of cancer cells were influenced by ADSCs. BALB/c mice were injected 5 × 10^5^ 4T1 or 1.5 × 10^5^ CT26 cancer cells with ADSCs subcutaneously. The tumor growth rate was evaluated by bioluminescent imaging and volume calculation. Representative images are shown in Figure 3D, and the results of tumor volume calculation are shown in Figure 3E. These data suggest that ADSCs significantly accelerate the growth rate of breast and colon cancer cells both in cell culture and in mice.

### The interaction of ADSCs with cancer cells increases the levels of IL-6 production

**Figure 4:**
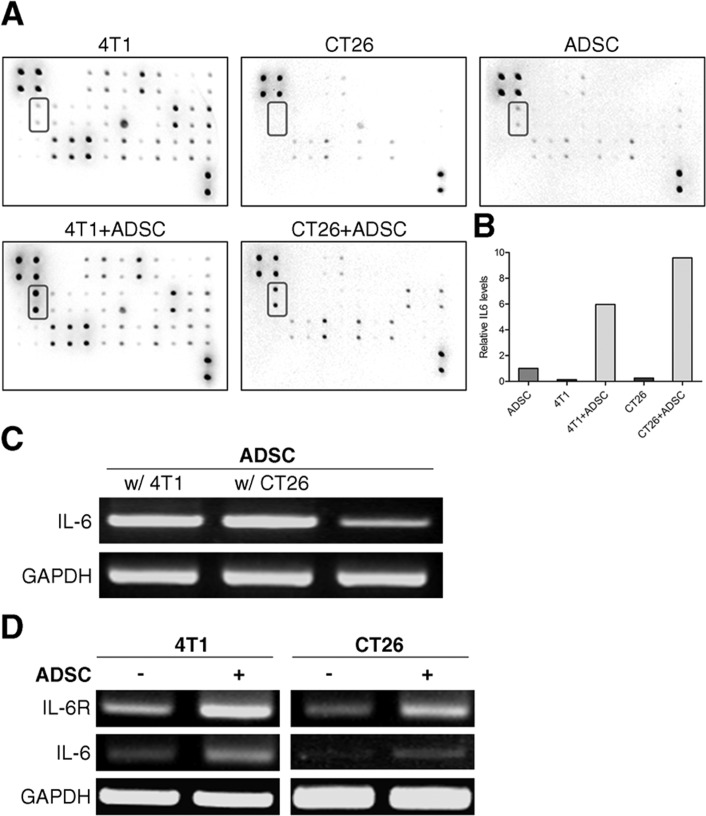
ADSCs interaction with cancer cells causes a rise in the levels of IL-6 (A) Cytokines profiles of cell-free culture medium from ADSCs, 4T1, ADSCs + 4T1, CT26, or CT26 + ADSCs were measured by RayBio mouse cytokine array 2. The highlighted box in the blot indicated the position of IL-6. (B) Quantitative analysis of the relative levels of IL-6 in cytokine array. Values are normalized to positive control and are relative to the levels of ADSC. (C) RT-PCR of *IL-6* expression in ADSCs alone or co-culture with 4T1 or CT26 cells, with *GAPDH* as loading control. (D) mRNA expression of *IL-6* and IL-6 receptor (*IL-6R*) in 4T1 or CT26 cells grown alone or in co-culture with ADSCs were evaluated by RT-PCR, *GAPDH* served as loading control.

Above results indicate that the pro-tumorigenic impacts of ADSCs may mediated by paracrine effects. We then determined the factor by which ADSCs utilized to enhance tumor initiation and growth of cancer cells. The differences of cytokine expression between cancer cells, ADSCs, and cancer cells plus ADSCs were examined by mouse cytokine array. The location of various cytokine capture antibodies spotted onto the RayBio Mouse cytokine antibody array kit was shown in Supplementary Figure S1. The dots on array film showed that the levels of IL-6 were almost undetectable in 4T1 or CT26 cells alone. IL-6 was also expressed low levels in ADSCs. However, the secretion of IL-6 was markedly increased in cultures of 4T1 plus ADSCs and CT26 plus ADSCs (Figure 4A). The relative expression level of IL-6 was calculated (Figure 4B). We further utilized RT-PCR to determine whether IL-6 is expressed by ADSCs. As shown in Figure 4C, ADSCs markedly upregulatd the mRNA level of IL-6 upon co-culture with 4T1 or CT26 cells. Interestingly, after co-culture with ADSCs both 4T1 and CT26 cells upregulated the expression of IL-6 receptor and slightly increased IL-6 expressions (Figure 4D). These results reveal that ADSC interaction with cancer cells could stimulate increased secretion of IL-6 mainly from ADSCs.

### IL-6 mediates the pro-tumorigenic impacts of ADSCs on breast and colon cancer cells

**Figure 5:**
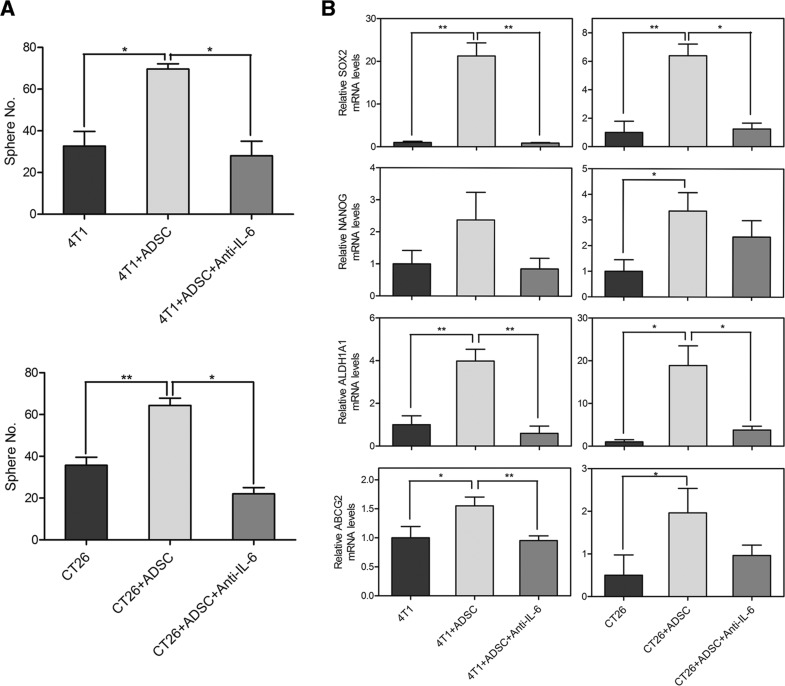
Involvement of IL-6 in ADSC-induced tumor-initiating properties of breast and colon cancer cells (A) Quantitation of spheres generated by 4T1 or CT26 cells co-cultured with or without ADSCs. Neutralizing antibody against IL-6 (Anti-IL-6) was additional added to the co-cultures of ADSCs with cancer cells. Values are means SEM; *, P<0.05; **, P<0.01 in unpaired t-tests with Welch's correction. (B) mRNA levels of CSC markers were determined by RT-PCR in 4T1 or CT26 cells with different conditions. Values (means + SEM) indicate relative mRNA levels compared to 4T1 or CT26 alone respectively after normalization to *GAPDH* loading control; *, P<0.05; **, P<0.01 using paired t-tests.

**Figure 6:**
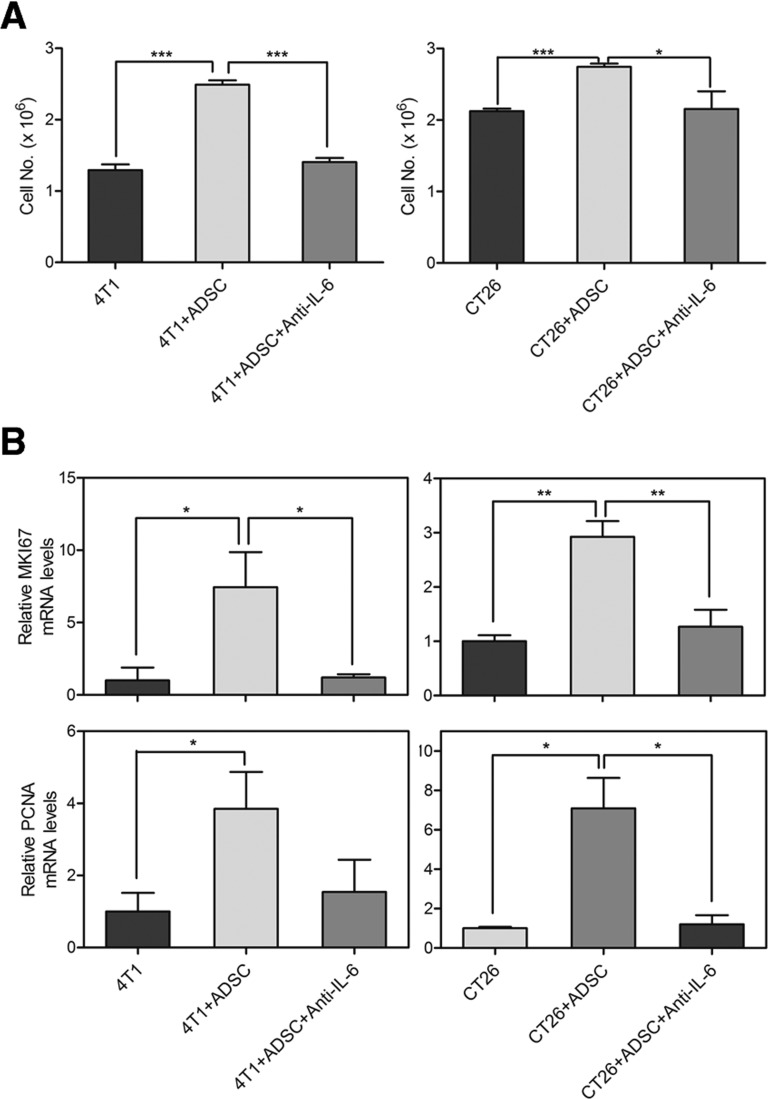
IL-6 mediates ADSC-induced growth of breast and colon cancer cells (A) Cell growth of 4T1 and CT26 cells grown alone or in co-culture with ADSCs was assessed by cell counting. Neutralizing antibody against IL-6 (Anti-IL-6) was additional added to the co-cultures of ADSCs with cancer cells. Values are means + SEM; *, P<0.05; ***, P<0.001 in unpaired t-tests with Welch's correction. (B) mRNA levels of *MKI67* and *PCNA* were determined by RT-PCR in 4T1 or CT26 cells with different conditions. Values (means + SEM) are normalized to *GAPDH* loading and are relative to levels of 4T1 or CT26 alone respectively; *, P<0.05; **, P<0.01 using paired t-tests.

To determine the contribution of IL-6 in mediating the ADSC-enhanced malignant characteristics of cancer cells, we utilized antibody to neutralize the function of IL-6. First, we examined the effects of IL-6 on tumor-initiating properties of breast and colon cancer cells. Parallel to the previous results, the number of tumor sphere was increased when co-cultured with ADSCs. But the ADSC-enhanced sphere generation of breast and colon cancer cells was significantly reduced by IL-6–neutralized antibodies (Figure 5A). We then utilized semiquantitative RT-PCR analysis to determine the effects of IL-6–neutralized antibodies on CSC markers. The expressions of self-renewal-related genes, including *SOX2* (4T1, 21.26 ± 3.06; CT26, 6.39 ± 0.82) and *NANOG* (4T1, 2.38 ± 0.86; CT26, 3.36 ± 0.72), and drug resistance-related genes, including *ALDH1A1* (4T1, 3.98 ± 0.55; CT26, 18.9 ± 4.59) and *ABCG2* (4T1, 1.55 ± 0.15; CT26, 1.96 ± 0.57), were relatively increased in cancer cells after co-culture with ADSCs. As expected, IL-6–neutralized antibodies markedly diminished the ADSCs-induced up-regulation of these genes in cancer cells, including *SOX2* (4T1, 0.87 ± 0.12; CT26, 1.24 ± 0.41), *NANOG* (4T1, 0.85 ± 0.33; CT26, 2.35 ± 0.64), *ALDH1A1* (4T1, 0.6 ± 0.34; CT26, 3.8 ± 0.88), and *ABCG2* (4T1, 0.95 ± 0.08; CT26, 0.96 ± 0.24) (Figure 5B). We then evaluated the effects of IL-6 on the growth abilities of cancer cells. As shown in Figure 6A, the ADSC-enhanced cell growth of cancer cells was significantly suppressed by IL-6–neutralized antibodies. Similar to the results of CSC marker genes, semiquantitative RT-PCR analysis showed that ADSCs strongly upregulate the growth-related genes in cancer cells, including *MKI67* (4T1, 7.44 ± 2.43; CT26, 2.93 ± 0.29) and *PCNA* (4T1, 3.85 ± 1.02; CT26, 7.09 ± 1.54). We also found that IL-6 neutralization markedly diminished the up-regulation of *MKI67* (4T1, 1.21 ± 0.22; CT26, 1.27 ± 0.31) and *PCNA* (4T1, 1.54 ± 0.9; CT26, 1.20 ± 0.46) induced by ADSCs in cancer cells (Figure 6B). Collectively, these results indicate that IL-6 as a key regulator that contributes to the ADSCs-induced enhancement of tumor-initiating properties and growth abilities in both breast and colon cancer cells.

### ADSCs activate JAK2/STAT3 in breast and colon cancer cells through IL-6 secretion

**Figure 7:**
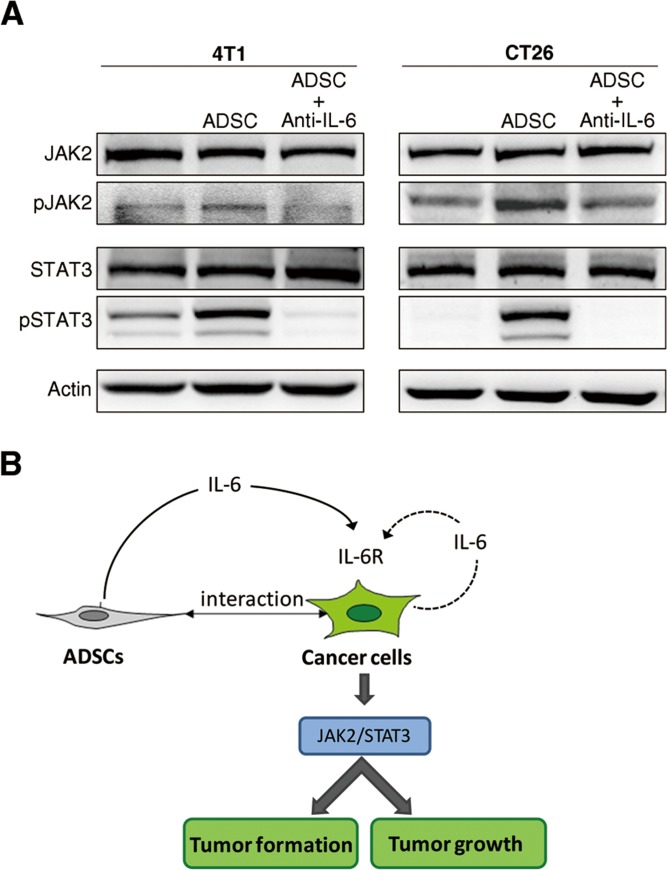
Activation of IL-6-dependent pathway in breast and colon cancer cells upon co-culture with ADSCs (A) Protein level of phosphorylated JAK2 and STAT3 in 4T1 and CT26 cells upon co-culture with ADSCs and treated with IL-6 neutralizing antibody (Anti-IL-6), with actin as loading control. (B) A schematic showing that IL-6 mediates the tumor-promoting effects of ADSCs in tumor development.

Above results suggest that ADSC-derived IL-6 played a critical role in activating malignant characteristics of cancer cells in cancer/ADSC interaction. To identify what downstream signals in cancer cells respond to IL-6, we looked at JAK2/STAT3 pathway. JAK2/STAT3 have been reported to be the predominant pathway activated by IL-6 [27]. As shown in Figure 7A, the protein levels of phosphorylated JAK2 and STAT3 in cancer cells were increased upon co-culture with ADSCs. Nonetheless, ADSCs-induced phosphorylations of JAK2 and STAT3 were inhibited by treatment with IL-6–neutralized antibodies. Together these results demonstrate that upon co-culture with cancer cells ADSCs can activate the JAK2/STAT3 pathways in both breast and colon cancer cells via induced IL-6 expression.

## DISCUSSION

It is well documented that various types of cells within the tumor microenvironment contribute to the development of cancer. Among these cells, MSC plays a critical role during tumor development. MSCs are multipotent cells that can self-renew and differentiate into various somatic lineages that contribute to the maintenance and regeneration of a variety of tissues, including bone, adipose tissue, cartilage and muscle [1]. The knowledge of the biological impacts of MSCs on cancer has been greatly improved recently. However, the underlying effects of MSCs on tumor development remain controversial, especially when MSCs come from different sources. MSCs with regenerative properties have been isolated from various types of tissue such as bone marrow, umbilical cord blood, placenta, and adipose tissue [3]. Among these tissues, adipose has been recognized as a rich source of adult stem cells. Compared to bone marrow, the common source of MSCs, it is less invasive and expensive to obtain stem cells from adipose tissue. Thus, ADSCs have attracted a lot of interest recently due to their convenient acquisition and regenerative capability. In addition, ADSCs have already applied in several medical fields such as plastic, orthopedic, and cardiac surgery. However, recent reports imply a potential tumorigenic role that ADSCs may play during tumor development, raising the concerns of their safety in clinical application [9, 19-20, 22]. Here, we showed that ADSCs promoted not only tumor-initiating capacity but also tumor growth ability of breast and colon cancer cells through IL-6-related pathway.

Tumor development is a multistep phenomenon, including tumor initiation, promotion, and progression. CSCs attracted much attention in the field of cancer research in recent years. They have been proved to play an important role in tumor initiation, invasion, metastasis, and resistance to anticancer therapies. BMSCs regulated CSC properties in several cancer types via various cytokines and chemokines [28-30]. Here we showed that ADSCs enhanced sphere generation and *in vivo* tumor formation of both 4T1 and CT26 cells, suggesting that ADSCs could facilitate the tumor-initiating capacity of breast and colon cancer cells. In addition to tumor initiation, we also demonstrated that ADSCs accelerated the tumor growth either in breast cancer or in colon cancer. The pro-growth effects of BMSCs on cancer have been widely demonstrated [31-33]. Recent reports led to similar conclusions that ADSCs promoted cancer growth and metastasis [8-11]. Moreover, Eterno *et al.* found that ADSCs could enhance breast tumor self-renewal and facilitate breast cancer recurrence via HGF/c-Met axis [21]. Although some conflict reports showed that ADSCs might exhibit anti-tumorigenic effects [12-14], we demonstrated that ADSCs play a promoting role during tumor development, especially in tumor initiation.

Breast and colon cancers are the second and third most common cancer in 2012 [23], and both on the rise worldwide. The incidence of breast and colon cancers has been proved to be highly associated with obesity [24-25]. Moreover, many reports revealed that obesity is linked to poor prognosis in breast and colon cancers such as reduced disease-free survival and overall survival [34-35]. Obesity may affect cancer progression and prognosis through numerous pathways, including modulation of energy balance and calorie restriction, hormonal influences, and inflammatory processes [36]. Mechanisms underlying the relationship between obesity and cancer are not fully understood. Nonetheless, overweight and obesity are highly related to alterations in the physiological role of adipose tissue, leading to insulin resistance, chronic inflammation, and changed secretion of adipokines. Accumulating evidence suggests that obesity could change the physiological characteristics of ADSCs such as enhanced proliferation and increased their number in fat tissue [16-17]. Recently, Strong *et al*. further identified that obesity could significantly alter the biological properties of ADSCs and these alterations enhanced the xenograft tumor growth of breast cancer cells [18]. In this study, we identified that ADSCs improved the malignant characteristics, including tumor growth and especially tumor initiation, of breast and colon cancer cells by secreting IL-6. Thus, ADSC stimulation may be an alternative mechanism by which dysfunctional adipose tissue promotes tumor development.

MSCs secrete various cytokines and chemokines that promote tumor development via paracrine- and/or autocrine-mediated pathways. MSCs also reveal direct impacts on tumor cells or indirect effects by modulating the surrounding tissue called the tumor microenvironment. Here, we found that IL-6 contributed to the protumor effects of ADSCs on tumor development in cancer cells by regulating genes that mediated tumor initiation and cell proliferation. IL-6 is a proinflammatory cytokine with multifunctions that exhibit wide range of biological activities. IL-6 has been related to many cancer types with a typical pro-tumorigenic effect. It has been shown to contribute to tumor development, including promotion of initiation [28-29], proliferation [37-38], and metastasis [39-40] and inhibition of apoptosis [37, 41], by binding to IL-6 receptor and co-receptor glycoprotein 130 (gp130), thus activating the downstream signaling pathway of the Janus protein tyrosine kinases (JAKs) and signal transducers and activators of transcription (STATs) [42]. Increased serum levels of IL-6 have been observed in a variety of cancers and predict an adverse outcome [43]. Furthermore, IL-6 is one of the major adipokines involved in adiposity-related inflammation and metabolic disease [44]. Several lines of evidence suggest that chronic inflammation contributes to tumor development at all three steps: tumor initiation, proliferation and progression. Thus, IL-6 may play an important role in the contribution of ADSCs to obesity-related cancer incidence.

In conclusion, we demonstrated that ADSCs play a pro-malignant role in tumor development of breast and colon cancer cells. Interaction of ADSCs and cancer cells stimulated secretion of IL-6 in ADSCs, which in turn acted in a paracrine manner on cancer cells to enhance their malignant properties, including tumor initiation (formation) and promotion (growth). IL-6 contributed to upregulation of genes related to CSCs and cell proliferation as well as activation of JAK2/STAT3 in cancer cells (Figure 7B). Much work yet needs to be done to fully understand the precise mechanisms of ADSCs facilitating tumor development. However, our findings, for the first time, suggest ADSCs can promote tumor initiation in tumor development. The results of current study are important to safety concerns regarding the clinical application of ADSCs. These results also suggest that ADSCs may contribute to obesity-related cancer incidence.

## MATERIALS AND METHODS

### Cell lines and ADSCs isolation

4T1 (ATCC CRL-2539) breast cancer cells and CT26 (ATCC CRL-2638) colon cancer cells were infected with FUW-Luc-mCh-puro [45] lentiviral particles and cultured in RPMI 1640 medium supplemented with 10% fetal bovine serum (FBS), 100 units/mL penicillin, 100 μg/mL streptomycin, 0.25 μg/mL amphotericin B, and 2 μg/mL puromycin in a humidified atmosphere with 5% CO_2_ at 37^o^C to stably express mCherry fluorescent protein. ADSCs were isolated and cultured as previously described [46]. Briefly, adipose tissues were obtained from abdominal cavity of mice and digested in αMEM containing 10% FBS and 0.1% collagenase type IV solution at 37^o^C for 1 hour. After ﬁltration through 45 μm nylon filter mesh (BD Falcon) and centrifugation for 10 min at 1500 rpm to remove ﬂoating adipocytes, the pellet (ADSCs) was resuspended in αMEM supplemented with 20% FBS, 100 units/mL penicillin, 100 μg/mL streptomycin, and 0.25 μg/mL amphotericin B and cultured in a humidified atmosphere with 5% CO_2_ at 37^o^C.

### Animal studies

All animal studies were approved by the Institutional Animal Care and Use Committee of Taipei Medical University. Four- to six-week-old female BALB/c or enhanced green fluorescence protein (EGFP) mice [FVB/NCrl-Tg(Pgk1-EGFP)3Narl], which ubiquitously expressed EGFP in all tissues, were purchased from National Laboratory Animal Center and National Applied Research Laboratories (Taipei, Taiwan). The mice were housed under pathogen-free conditions and fed autoclaved food and water. For *in vivo* tumor formation experiments, 6 × 10^3^ 4T1 cells or 3 × 10^4^ CT26 cells were implanted with or without five folds of BALB/c ADSCs via subcutaneous injection. For *in vivo* tumor growth experiments, 3 × 10^5^ 4T1 cells or 1.5 × 10^5^ CT26 cells were implanted with or without five folds of BALB/c ADSCs via subcutaneous injection.

### Cell co-culture studies

For direct co-culture, 1 × 10^5^ cancer cells or 1 × 10^5^ cancer cells plus 5 folds of BALB/c ADSCs were seeded into each well in 6-well plates using 3 wells/cell line. After 3 days of co-culture, cells were harvested and conducted with *in vitro* BLI assay to evaluate the amount of cancer cells. For indirect co-culture, cells were co-cultured by using transwell inserts with a 0.4-μm porous membrane (Corning) to separate ADSCs from cancer cells. 5 × 10^5^ BALB/c ADSCs were seeded into upper chamber and 1 × 10^5^ cancer cells were seeded into lower well. After co-culture for 3 days, cancer cells were harvested and counted. The indirect co-culture of ADSCs and cancer cells was also conducted for RT-PCR and western blot assays. For IL-6 neutralization assay, ADSCs and cancer cells were indirectly co-cultured by using transwell inserts with additional adding of 0.25 μg/mL IL-6 neutralizing antibody.

### Flow cytometry

For cell surface marker analysis, cells were harvested, resuspended in 100 μL HBSS containing specific antibodies, and incubated at 4°C for 30 min. Samples were analyzed using FACSCanto II low cytometer (BD Biosciences) and FCS Express software (De Novo). Antibodies were used at concentrations as recommended by the manufacturer. Antibody information is provided in Supplementary Table S1.

### Reverse transcription-PCR

Total RNA was extracted from cells using High Pure RNA Isolation Kit (Roche) according to the manufacturer's instructions. Reverse transcription-PCR was performed as previously described [47]. Primer sequences are listed in Supplementary Table S2. Relative quantitation was performed using ImageJ software (Version 1.46r).

### Multilineage differentiation assays

To evaluate the *in vitro* differentiation potential of cells, we conducted differentiation induction experiments of three major mesodermal lineages. Briefly, cells were seeded in 6-cm tissue culture dishes to 80-90% confluence. For osteogenic differentiation, cells were cultured in α-MEM supplemented with 10% FBS, 0.1 μM dexamethasone, 10 mM β-glycerophosphate, and 50 mM ascorbic acid for 21 days, and cells were stained with 2% Alizarin Red S (pH 4.2) for 15 min at room temperature. For adipogenic differentiation, cells were cultured in α-MEM supplemented with 10% FBS, 1 μM dexamethasone, 10 μg/mL insulin, and 0.5 mM 3-methyl-1-isobutylxanthine for 2 days, then cells were incubated for 21 days in maintenance medium (α-MEM, 10% fetal calf serum, and 10 μg/mL insulin). Cells were fixed and stained with 0.5% oil red O in 60% isopropyl alcohol for 15 min to detect lipid droplets. For chondrogenic differentiation, cells were cultured in α-MEM supplemented 10% FBS, 10 ng/mL TGF-β1, 10 nM dexamethasone for 21 days, and stained with 1% Alcian blue 8GX reagent in 3% glacial acetic acid (pH 2.5) for 30 min at room temperature.

### Sphere formation assay

To observe living cells in tumor spheres with fluorescence microscope, we isolated ADSCS from EGFP mice. 4 × 10^4^ ADSCs, 4 × 10^4^ cancer cells, or 4 × 10^4^ cancer cells plus 2 × 10^5^ ADSCs were incubated for 14 days in 4 mL of modified sphere medium (DMEM/F12 medium supplemented with 1X B-27 supplement (Gibco), 20 ng/mL epidermal growth factor (PeproTech), 10 ng/mL fibroblast growth factor-basic (PeproTech), and 20 ng/mL human leukemia inhibitory factor (Sigma-Aldrich) in T25 flasks. Spheres (>50 μm diameter) in ten random fields per each flask were counted and photographed.

### Bioluminescence imaging (BLI)

BLI of cells and animals was performed with an IVIS Imaging System 200 Series (PerkinElmer) and quantitated with Living Image® software by measuring photon flux (photons/s/cm2/steradian) in regions of interest drawn around appropriate signals. For *in vitro* BLI, cells were treated with cell lysis buffer (Promega) and placed in 96-well black imaging plates, and 100 μL D-Luciferin reagent (1.5 mg/mL) (Gold Biotechnology) were added to each well and mixed well, and 30 sec later BLI was performed and the signal was acquired for 1 min. For *in vivo* BLI, anesthetized mice were injected intraperitoneally with 75 mg/kg of D-Luciferin and images were acquired 2–5 min after injection. Acquisition times were 2 min initially and were reduced in accordance with signal intensity to avoid saturation.

### Cytokine array

Cells were cultured in complete media for 3 days. The levels of cytokines, growth factors and chemokines in the culture media were assessed by RayBio® Mouse Cytokine Antibody Array 2 (#AAM-CYT-2) according to the manufacturer's instructions. Chemiluminescent data was collected using the Multi-function Gel Image system (TOP BIO CO., MultiGel-21) and quantitated with ImageJ software (Version 1.46r) by measuring the intensities of detected spots.

### Western blot analysis

The protein extraction and immunoblotting were performed as previously described [48]. Antibody information is provided in Supplementary Table S1.

### Statistical analysis and replicates

The sizes of sample group in all data are at least n = 5, unless otherwise indicated. All data presented are representative of at least three independent experiments that yielded similar results. Statistical analyses were performed using GraphPad Prism 5.

## SUPPLEMENTARY MATERIAL, FIGURES, TABLES


