# Supplementary material for: Adipose-derived stem cells promote tumor initiation and accelerate tumor growth by interleukin-6 production
Source: Oncotarget. 2015 Mar 8;6(10):7713–26. doi: 10.18632/oncotarget.3481 (PMC4480711; doi:10.18632/oncotarget.3481)
Supplement: Supplementary file 1 [file oncotarget-06-7713-s001.pdf]

Adipose-derived stem cells promote tumor initiation and accelerate tumor growth by interleukin-6 production

Supplementary Material

Supplementary Table S1: List of antibodies used in this study

Antibody name	Application	Company & Cat.No.	Conditions used
Anti-Mouse CD34 PE	Flow cytometry	BioLegend #119307	0.25 µg/test
Anti-Mouse CD45 PE	Flow cytometry	BioLegend #103105	0.25 µg/test
Anti-Mouse CD90 PE	Flow cytometry	BioLegend #140307	0.25 µg/test
Anti-Mouse CD105 PE	Flow cytometry	BioLegend #120407	0.25 µg/test
Anti-Mouse Sca-1 PE	Flow cytometry	BioLegend #108107	0.25 µg/test
Rat IgG2a K Isotype Control PE	Flow cytometry	BioLegend #400507	0.25 µg/test
Rat IgG2b K Isotype Control PE	Flow cytometry	BioLegend #400607	0.25 µg/test
Rabbit monoclonal anti-JAK2	Western blot	Cell Signaling Technology #3230	1:1000
Rabbit polyclonal anti-phospho-JAK2	Western blot	Cell Signaling Technology #3771	1:500

Rabbit polyclonal anti-STAT3	Western blot	GeneTex #GTX110587	1:1000
Rabbit monoclonal anti-phospho-STAT3	Western blot	Cell Signaling Technology #9145	1:1000
Mouse monoclonal anti- Actin	Western blot	Millipore #MAB1501	1:10000
LEAF™ Purified anti-mouse IL-6 Antibody	IL-6 neutralization	BioLegend #504506	0.25 μg/mL

Supplementary Table S2: List of primer sequences used in RT-PCR

**Target gene**	**Forward primer**	**Reverse primer**
PPARG	5’-CACTTCACAAGAAATTACCAT-3’	5’-GAAGGACTTTATGTATGAGTC-3’
Leptin	5’-TGCTCCAGATAGCCAATGAC-3’	5’-GAGTAGAGTGAGGCTTCCAGGA-3’
OPN	5’-ATGAGATTGGCAGTGATT-3’	5’-GTTGACCTCAGAAGATGA-3’
RUNX2	5’-ACTTTCTCCAGGAAGACTGC-3’	5’-GCTGTTGTTGCTGTTGCTGT-3’
COL2A1	5’-GAACAGGAATTTGGTGTGGA-3’	5’-AGGTCCCCGTGGACGCTCAG-3’
ACAN	5’-GACCAGGAAGGGAGGAGTAG-3’	5’-CAGCCGAGAAATGACACC-3’
SOX2	5’-GCCTGGGCGCCGAGTGGA-3’	5’-GGGCGAGCCGTTCATGTAGGTCTG-3’
NANOG	5’-AGGGTCTGCTACTGAGATGCTCTG-3’	5’-CAACCACTGGTTTTTCTGCCACCG-3’
ALDH1A1	5’-AACTCCTCTCACGGCTCT-3’	5’-GCTCGCTCAACACTCCTTTTC-3’
ABCG2	5’-TTACCCTTATAATGGTGGCTTATACGG-3’	5’-CAAAGCTGTGAAGCCATATCGAG-3’
MKI67	5’-GGAGGCAATATTACATAATTTCA-3’	5’-CAGGGTCAGAAGAGAAGCTA-3’

PCNA	5’-GGTTGGTAGTTGTCGCTGTA-3’	5’-CAGGCTCATTCATCTCTATCG-3’
IL6	5’-ATGGATGCTACCAAACTGGAT-3’	5’-TGAAGGACTCTGGCTTTGTCT-3’
IL6R	5’-GCCCAAACACCAAGTCAAGT-3’	5’-CTCATTCCCTGAAGCAGAGC-3’
GAPDH	5’-GCTCTCCAGAACATCATCCCTGCC-3’	5’-CGTTGTCATACCAGGAAATGAGCTT-3’

	A	B	C	D	E	F	G	H	I	J	K	L
1	Pos	Pos	Neg	Neg	6Ckine	CTACK	Eotaxin	GCSF	GM-CSF	IL-2	IL-3	IL-4
2	Pos	Pos	Neg	Neg	6Ckine	CTACK	Eotaxin	GCSF	GM-CSF	IL-2	IL-3	IL-4
3	IL-5	IL-6	IL-9	IL-10	IL-12 p40p70	IL-12p70	IL-13	IL-17	IFN-γ	KC	Leptin	MCP-1
4	IL-5	IL-6	IL-9	IL-10	IL-12 p40p70	IL-12p70	IL-13	IL-17	IFN-γ	KC	Leptin	MCP-1
5	MCP-5	MIP-1α	MIP-2	MIP-3 β	RANTES	SCF	sTNFRI	TARC	TIMP-1	TNF-α	Thrombopoietin	VEGF
6	MCP-5	MIP-1α	MIP-2	MIP-3 β	RANTES	SCF	sTNFRI	TARC	TIMP-1	TNF-α	Thrombopoietin	VEGF
7	BLANK	BLANK	BLANK	BLANK	BLANK	BLANK	BLANK	BLANK	BLANK	BLANK	BLANK	Pos
8	BLANK	BLANK	BLANK	BLANK	BLANK	BLANK	BLANK	BLANK	BLANK	BLANK	BLANK	Pos

Supplementary Figure S1: Template showing the location of antibodies for various cytokines spotted onto the RayBio® Mouse Cytokine Antibody Array 2.
